# Bridging social and ecological science to create spatially explicit models of human-caused mortality of carnivores

**DOI:** 10.1007/s13280-025-02165-1

**Published:** 2025-04-29

**Authors:** Jeremy T. Bruskotter, Neil H. Carter, Richard Berl, Joseph Hinton, Jazmin Murphy, L. Mark Elbroch, John A. Vucetich

**Affiliations:** 1 School of Environment and Natural Resources, The Ohio State University, 2021 Coffey Rd., 210 Kottman Hall, Columbus, OH 43210 USA; 2 School for Environment and Sustainability, University of Michigan, 440 Church St, Dana Building, Ann Arbor, MI 48109 USA; 3 Eastern Ecological Science Center, Patuxent Research Refuge, 12100 Beech Forest Road, Laurel, MD 20708 USA; 4 Wolf Conservation Center, 7 Buck Run St, South Salem, NY 10590 USA; 5 Wolf Conservation Center, University of California, Santa Barbara, 7 Buck Run St, South Salem, NY 10590 USA; 6 Panthera, 8 West 40th Street, 18th Floor, New York, NY 10018 USA; 7 College of Forest Resources and Environmental Science, Michigan Technological University, 1400 Townsend Drive, Houghton, MI 49930 USA

**Keywords:** Anthropogenic mortality, Behavioral science, Carnivore conservation, Human dimensions, Human-caused mortality, Habitat suitability

## Abstract

Research indicates that human-caused mortality (HCM) is a key factor limiting numerous large carnivore populations. However, efforts to represent HCM in spatially explicit models have generally been limited in scope—often relying on proxies, such as road or human density. Yet such efforts fail to distinguish different sources of HCM, which can arise from different antecedent processes. We offer a systems-based conceptual framework for understanding the antecedents of HCMs that is grounded in theory from the social and behavioral sciences. Specifically, we first explain how HCMs are usefully distinguished into four types (e.g., accidental, harvest, illicit, control actions), then discuss how these different types tend to be driven by different sets of psychological and sociopolitical processes. We contend that improvements in understanding the spatial variation in HCMs would rise from more explicit attention to the various antecedent processes that precede each mortality type.

## Introduction

As human impacts on organisms and their environments accelerate (Barnosky 2008; Ceballos et al. 2017), human activities are increasingly recognized as the key driver of range loss and extinction risk for large mammals (Cardillo et al. 2008; Ripple et al. 2014). This recognition has prompted the use of abiotic proxies for anthropogenic impacts on wildlife (e.g., road density, distance to human infrastructure) in efforts to predict, map, and manage human-wildlife conflicts and associated mortalities (e.g., Naves et al. 2003; Treves et al. 2004; Lamb et al. 2020; Van den Bosch et al. 2022). Ongoing refinements in anthropogenic indices (e.g., Global Human Impact Index) better capture the ‘footprint’ of humans (WCS 2005), and technological advances such as smartphone tracking have improved our ability to capture human activities (Korpilo et al. 2017). However, despite such advancements, efforts to project where anthropogenic mortalities are more likely to occur, with few exceptions (e.g., Gangaas et al. 2013; Gálvez et al. 2018; Manfredo et al. 2021), fail to account for the underlying processes that drive human behavior. A better understanding of the causal processes that lead to mortalities could increase the chance that subsequent management actions will result in their desired effects, such as reductions in mortality.

In the case of large-bodied, terrestrial carnivores (i.e., those weighing > 15 kg; Bruskotter et al. 2017), numerous studies demonstrate that human-caused mortality (HCM) is often the most important source of mortality for these populations (e.g., Hinton et al. 2017; Treves et al. 2017; Gantchoff et al. 2020; Agan et al. 2021; Breck et al. 2023)—even for many protected populations (e.g., Hebblewhite and Whittington 2020). Though HCMs are not always the primary source of mortality for large carnivores (hereafter “LCs”; e.g., Borg et al. 2015), conservation practitioners have long recognized HCM as a critical obstacle to conserving LCs (Mattson and Reid 1991; Fritts and Carbyn 1995; Creel et al. 2010).

Despite recognizing the importance of HCM, efforts to model mortality risk and other factors affecting LC populations generally still rely upon proxies of human presence and activity. In fact, a recent review of more than 1200 studies published from 1995–2021 found the most common anthropogenic covariates included in these models were distance metrics (e.g., distance to roads), density metrics (e.g., human population density) and land use metrics (e.g., proportion of area in agriculture)—these were represented in 27, 23, and 23 percent of studies, respectively (Titus and Jachowski 2025).

The concern is that HCMs do not result solely from relatively fixed conditions of the environment, such as distant from roads or human density. Rather they result from a combination of internal (i.e., human thought processes) and situational (e.g., societal and environmental conditions) factors that affect human behavior (Harré et al. 2015; Carter et al. 2017). If one desires to understand why HCMs occur, one first needs to understand the underlying system that produces these mortalities, including the psychological and societal processes that can promote or inhibit HCMs (Bruskotter et al. 2015; Carter et al. 2019). Such understanding is aided by extending the concept of an organism’s environment beyond the biotic and abiotic (Mitchell 2005; Kearney 2006) to include human behaviors and the institutions governing those behaviors in a socio-ecological system (Carter et al. 2017). Taking account of such phenomena in a socio-ecological framework could reveal spatial variability in social processes that help better characterize the risks of HCMs.

In the summer of 2023, a group of carnivore ecologists, ecological modelers and social scientists met for two days with one aim: to improve our collective understanding of where and why HCMs occur by considering how social science insights could be synthesized to improve spatially explicit models of HCM risk. To that end, we adopted a ‘systems’ approach that began with the taxonomization of different types (or sources) of HCM, and then sought to identify spatially explicit factors likely to affect the rate at which each type of HCM occurs. Subsequently, we refined the initial list of factors iteratively by considering (a) The importance of each variable as a potential cause of the human behaviors that promote or inhibit HCMs, and (b) The potential for quantifying each factor in a spatial unit of appropriate scale. Note, although we did not specifically limit our efforts to any taxonomic group or geographic region, our collective research experience emphasizes the families Canidae and Felidae, primarily in the Americas. In what follows, we present the results of this process, which, with some adaptation, should be useful across a wide range of species and geographic regions. Our hope is that this effort will begin a conversation concerning how the social sciences can better contribute to a variety of spatially explicit conservation models.

## Taxonomizing sources of human-caused mortality: a first step

The first step in identifying the processes that give rise to HCMs is to distinguish *at least* four types of HCMs: accidental, harvest, illicit, and control actions (Table 1). Critically, differences in the processes giving rise to these types of HCM mean that the risk of one mortality type may be independent from others, as we explain below.

**Table 1:** Four common types of human-caused mortalities for large carnivores

Human-caused mortality type	Definition
Accidental	Unintended mortalities either directly or indirectly caused by humans. Many instances of ‘road kill’ would be examples
Harvest	Intentional mortalities that do not violate existing law and tend to be characterized by extensive regulatory limitations on where, when and how these mortalities occur
Illicit	Intentional mortalities that violate existing law or, where formal law is absent, prevailing social norms
Control Action	Intentional mortalities that occur in response to some specific harm (e.g., killing of a pet or livestock) caused by the species or individual animal being controlled. These actions are generally performed by some government-sanctioned agent

Elaborating on Table 1, *accidental mortalities* include incidents where humans directly cause mortality events (e.g., vehicle collisions) as well as mortalities that are indirectly caused by humans, such has those due to our infrastructure (e.g., an animal falls into an abandoned mine) or other habitat modifications. Importantly, because they are unintentional—the key feature distinguishing them from other mortality types—we expect that, in most case, accidental mortalities will be primarily driven by institutional processes (e.g., zoning, road-building, etc.) generally governed by bureaucratic agencies (e.g., zoning boards, Departments of Transportation).^1^ These processes generally determine where human infrastructure appears.

With respect to *harvest mortalities* (Table 1), research indicates recreational hunting and trapping can involve a variety of underlying motivations (e.g., Decker and Connelly 1989; Schroeder et al. 2018). In the case of carnivores, one might shoot an animal primarily for recreational enjoyment while harboring no hostility toward that animal specifically, nor the species more generally. Alternatively, one might hunt because one perceives a population to be over-abundant or out of retribution for harms caused by animals belonging to the hunted species (Schroeder et al. 2018). A key feature of harvest mortalities is that they are regulated by law, which affects both location and timing (e.g., limited to certain times of year, or daylight hours) as well as the types of equipment that may be used (e.g., bans on foothold traps). Indeed, hunting and trapping are often prohibited by municipal governments in urban or suburban areas (Jones and Rodriguez 2003); thus, we would not expect harvest mortalities to occur in such locations, whereas accidental mortalities may be more likely in those locations.

Like harvest mortalities, *control actions* are intentional; however, unlike harvest mortalities, control actions are generally carried out by a third party (e.g., government agent) and justified by some perceived management goal, such as removing animals suspected of killing livestock (Linnell et al. 2012), or reducing predation on wild ungulate (prey) populations (Hervieux et al. 2014). Control actions differ from other types of HCM in that the initial encounter that elicits an action may or may not directly involve a human. For example, consider a scenario where a livestock producer finds a dead calf and evidence (e.g., tracks, bite marks) suggesting it was killed by gray wolves (*Canis lupus*). The producer calls a government agency that sends a trapper who eventually traps and kills a wolf in the vicinity of the dead calf. In this scenario, the individual ultimately responsible for the control action (the producer) might never interact with the offending animal; rather, the interaction that motivates the event is with the livestock producer’s property.

The fact that the ‘encounter’ that leads to a control action does not require the presence of a human is an important distinguishing feature of control actions, as the presence of property such as livestock may be unrelated (or even negatively related) to normal indices of anthropogenic activity (e.g., road or human density). Imagine, for example, a calf killed on public lands managed by the U.S. Forest Service—lands generally far from human development. Because of their distance from human settlement, these types of protected natural areas are often considered to be refugia for LCs (e.g., Wolf and Ripple 2017). However, policy in the USA that allows for public lands grazing puts livestock and carnivores in proximity, potentially increasing the risks to both species (Hanley et al. 2018).

Control actions are generally governed by regulations that differ from those governing hunting or trapping. For example, they may allow for the use of tools that are prohibited for hunters, such as poison baits or electronic calls, and may occur during times of the year in which legal harvests are prohibited.

Control actions can be characterized as resulting from both the internal thought processes of some affected person (e.g., livestock producer) and their interactions with a regulatory agency or other third party that typically performs or approves the control action. Moreover, that agency may provide additional recommendations about how future problems can be avoided (e.g., non-lethal deterrents) thereby reducing the risk of future HCMs.

Finally, *illicit* mortalities are a third form of intentional killing—but their distinguishing feature is that they violate existing law or, where formal law is absent, prevailing social norms. Because illicit killing violates law (or prevailing social norms), those who engage in such killing have an incentive to hide their activities in a way that can influence when and where they occur (e.g. Aziz et al. 2017), thereby differentiating it from legal harvest. For example, poachers may choose locations further from human settlements (e.g., public lands or large private properties) to hide their activities from authorities (Ghoddousi et al 2022). Likewise, illicit killing may involve tools that are not necessarily available to those who hunt legally (e.g., poisons, traps), which may affect both where and when they occur.

Because they require *intent*, it may be tempting here to characterize instances of illicit mortality as acts of intolerance (some scholars define intolerance as behavioral acts intended to harm species; see Bruskotter and Fulton 2012). However, such a view would not account for the multiple types of motivations for killing wildlife (Decker and Connelly 1989). For example, animals or animal parts that fetch high dollar values may bring out poachers who are motivated by financial interests (Lunstrum and Givá 2020). Yet, illicit killing might also be motivated by the desire to eliminate animals seen as a danger to human interests or livelihoods, retribution for prior harm (e.g., killing livestock), overt threats to human safety, or even as a form of rural resistance (see: Treves and Bruskotter 2014; Kahler and Gore 2015; Von Essen et al 2015).

Our brief review of these four sources of HCM illustrates that these types of HCMs should vary with respect to their spatial and temporal distributions. Though we attempted to be exhaustive in our typology, we acknowledge that other types of HCMs might exist in cultural contexts with which we are not familiar. Likewise, the types of HCMs we detail are not strictly mutually exclusive. One might, for example, illegally shoot and kill a legally-protected red wolf believing it to be a coyote. Our purpose in distinguishing among possible types of HCM is to serve our next focus, which is to assess how the antecedents of HCMs can vary across these types—and an adequate understanding of HCM requires explicitly attending those various antecedents (see also Wilkinson et al. 2020).

## Identifying antecedents of human-caused mortalities

All types of HCMs share one commonality—they begin with an encounter that occurs when an animal and a human, or some stand-in agent for a human, such as a trap or livestock, co-occur in space and time (Carter et al. 2017; see Table 2). Such encounters often are attributable to anthropogenic food sources (e.g., trash for black bears; Lewis et al. 2015). Each encounter effectively presents an opportunity for a mortality event to occur. Accidental mortalities require only an encounter and so can be contrasted with harvest and illicit mortalities which also require *intention* to kill or otherwise harm an individual animal. To elaborate, though hunters and poachers alike may be motivated by a wide range of factors that vary across species and contexts, the behavioral actions required to kill an individual animal require one’s intent (and in many instances, extensive preparation). Yet, intent alone is insufficient for killing an animal. Indeed, even in instances where one intends to pursue and kill an animal, each actor will be limited by their *capacity* to carry out behavioral acts that result in an animal’s death (Bruskotter and Fulton 2012; Carter et al. 2017).^2^ These three elements (i.e., intention, capacity, opportunity) are all either properties of an individual (in the case of intention and capacity) or at least somewhat under their volitional control (in the case of opportunity) and thus will vary considerably across persons, and potentially, across spatial units.

**Table 2:** Human-caused mortalities for carnivores: sources and antecedent processes

Mortality type	Antecedent processes				
	Individual level			Social-contextual level	
	Opportunity (Encounter)	Capacity	Intention	Guardianship	Institutional facilitation
Accidental	R				X
Harvest	R	X	R		X
Illicit	R	X	R	X	X
Control Action^a^	R		R	X	R

X—indicates an antecedent process (columns) affects the probability of the specific mortality source (row)

R—indicates an antecedent thasemantics in the study oft is required for a particular type of mortality to occur

^a^Control actions are generally conducted by external agents. Although agents employed to remove carnivores should have the capacity to do so, they are typically called upon precisely because the affected individual lacks this capacity

Two additional processes external to the individual may also affect HCMs, especially those motivated by intolerance for an animal. Following Carter et al (2017), we call the first element, *guardianship*. Guardianship represents the likelihood that other humans will attempt to report or otherwise thwart an instance of killing. While, in principle, guardianship is external to the individual, it is likely to impact an individual’s behavior when it is perceived by that individual as a normative prohibition against killing or harming individuals of the species in question (Steinmetz et al. 2014). In such cases, we anticipate that when an individual human perceives that they are likely to be sanctioned for killing an animal, this perception could impact their behavior both directly (e.g., despite intending to commit an act of intolerance they exhibit restraint during an encounter) or indirectly, by affecting their intention (for a review of the role of intention in predicting behavior generally, see, Ajzen and Kote, 2008).

The second element, which we refer to as *institutional facilitation,* represents the institutional equivalent of guardianship, and refers to the extent to which agencies charged with managing public resources (e.g., lands, roads, wildlife) create policies that facilitate or inhibit different sources of HCMs. Like guardianship, institutional facilitation is a process external to the individual that could conceivably impact other antecedent processes. For example, regulatory policies limit legal opportunities by governing the timing and duration of hunting seasons, as well as the location of hunts. Additionally, institutional facilitation may also affect mortalities without impacting other antecedent elements, as when agencies adopt policies promoting control actions designed to reduce populations (e.g., killing coyotes (*Canis latrans*) and mountain lions (*Puma concolor*) to aid mule deer; Hurley et al. 2011).

Moreover, though an objective of harvest policy (one type of institutional facilitation), in most instances, is to limit human-caused mortality, it is possible that such limitations could have unintended consequences. To illustrate, consider two social contexts: in the first, there is very strong normative pressure among human hunters against poaching of wolves; in the second, the situation is reversed—poaching is socially supported by local hunters. In the first context we might anticipate that increasing opportunities for hunting white-tailed deer (*Odocoileus virginianus*) would have little effect on poaching of wolves, as it is deemed unacceptable behavior. In contrast, in the second context lengthening the legal season for deer may inadvertently facilitate poaching of wolves by increasing opportunities (more time in the field) for poaching among those not constrained by normative pressures. Moreover, normative pressure may differ across groups of people. Thus, if deer hunters believed illegal killing of wolves was not only acceptable, but desirable, it may lead to increased instances of poaching regardless of season limitations.

This hypothetical serves to illustrate how changes in policies, even when those policies are directed at another species, can have unintended consequences—in this case, increasing poaching. It also illustrates how the effect of one type of antecedent process may be dependent on another—in this case, the effect of institutional facilitation depends upon social norms (guardianship).

## Toward a theoretical framework for explaining anthropogenic mortalities

Describing how key antecedent processes impact HCMs is aided by integrating theory from across the social and behavioral sciences—theory ultimately aimed at understanding and explaining human behavior. We turn next to briefly outline theories that offer insights into how best to conceptualize and measure the behavioral antecedents described above and discuss how these insights might be used to improve spatially explicit models of HCMs.

### Intention

Decades of psychological research indicate that an especially important predictor of volitional behavior (i.e., behavior under one’s control) is one’s *intention* to engage in that behavior (Fishbein and Azjen 1975, 2011), and this includes intentions to hunt/harvest wildlife (Hrubes et al. 2001). Clearly, hunting and trapping, whether undertaken in accordance with all local laws or not, involves intention. However, because poaching is, by definition, an illicit behavior, individuals may be motivated to misreport or conceal their intentions (for a review, see Von Essen et al. 2014). Fortunately, some developments in the measurement of illicit behavior could be useful for addressing such concealments (see: Gavin et al. 2010; Nuno and St. John 2015). Alternatively, one might use a proxy for intention, such as one’s general attitude toward a species. Indeed, Bruskotter et al. (2015) demonstrated that individuals’ general attitudes toward species (in this instance, wolves) were strongly associated with self-reports of both oppositional and supportive intentions directed at wildlife (i.e., tolerance). Moreover, subsequent research showed both attitudes and behaviors are strongly related with the perceptions of risk and benefits individuals associate with species (Carlson et al. 2023).

### Capacity

Conceptually, capacity represents the extent to which an individual can perform the necessary task(s) involved in finding and killing the target animal (for discussion, see Bruskotter et al. 2015). These tasks depend on the species being targeted, the method and tools used to find and kill the animal, and the skill and equipment necessary to carry out said tasks. Accurately discharging a firearm, for example, involves a different set of tasks and skills than appropriately setting a foothold trap. In the USA, it appears that the majority of illicit killings of LCs involve firearms (Hinton et al. 2017; Turnbull et al. 2013). In instances of firearms poaching, access to a firearm is (of course) necessary. Thus, one potential proxy for assessing spatial variation in capacity is the gun ownership rate within a particular area; others include hunting license purchase and overall harvest.

One’s capacity to poach via firearm is also affected by one’s skill with the weapon, and ability to track and find the animal one intends to kill. Therefore, we anticipate capacity to be higher in areas with higher rates of hunting and trapping because those individuals would have more experience practicing the necessary skills. In the USA, data on hunting and trapping license purchasing is maintained by the U.S. Fish and Wildlife Service, but only available at the state level. Depending upon the context, researchers might also be aided by developing a richer account of capacity, including, for examples, prior hunting experience or self-assessments of skills (e.g., marksmanship) that could be estimated via self-administered surveys. Finally, capacity can also be limited by institutional regulations, particularly those that reduce hunter lethality (e.g., bans on leghold traps).

Numerous behavioral theories assess factors analogous to capacity, such as ‘perceived behavioral control’ and ‘self-efficacy’ as predictors of behavior (see Ajzen 2002 for review). Though psychologists sometimes distinguish between these factors, the general insight emerging from such research is that engagement in a behavior is limited to the extent that one perceives the performance of a behavior as under one’s volitional control (Ajzen 2002). One recent study found that perceived behavioral control moderated the effect of intention (to engage in a behavior) on the actual performance of that behavior such that intention had a greater effect when one perceived greater control over the behavior (Hagger et al. 2022). Regardless, insights from research on perceived behavioral control and similar psychological phenomena could prove useful in guiding expectations about how one’s capacity to perform necessary behaviors affects estimation of both harvest and illicit mortalities.

### Opportunity

An opportunity to poach an animal arises as the result of an encounter—i.e., when a person (or their devices; e.g., trap, poison) and a wild animal co-occur in space and time (Carter et al. 2017). We expect the probability of such interactions will generally increase with both human and carnivore activity within an area. Researchers interested in the quality of carnivore habitat have long used road density as a proxy for humans’ opportunity to access (and kill) carnivores (e.g., Treves et al. 2004; Jędrzejewski et al. 2008). Moreover, some species (e.g., wolves) tend to utilize roads for travel (Kohn et al. 2009), thus increasing the probability of co-occurrence and, potentially, associated mortalities (Dennehy et al. 2021). At intermediate spatial scales (e.g., counties), human and road densities tend to be highly correlated (Karns et al. 2015), which suggests either variable might be useful. However, higher human densities also mean potentially more ‘guardians’ (individuals willing to report illegal killing), and so may be protective in instances where there are strong normative prohibitions against hunting and/or poaching. Finally, like capacity, opportunities may be limited by regulatory frameworks put in place by land or wildlife agencies (e.g., length of hunting seasons) and local municipalities (e.g., prohibition against discharging firearms).

### Guardianship

Eliason (2012) explained that the absence of capable guardians facilitates poaching. From this perspective, guardianship is largely a function of institutional enforcement activities (e.g., strong laws, number of law enforcement officers, frequency of patrolling). However, the expectation that one might be caught, prosecuted, and punished could also affect an individuals’ propensity to poach (Messer 2000), and should be based, in part, on the individual’s expectation of being sanctioned (Apel 2013). That is, in situations where one* perceives* a higher risk of being caught and penalized (sanctioned), we should expect poaching to be less frequent. In contrast, in areas where people do not anticipate sanctioning, one would expect poaching to occur with greater frequency.

The propensity to sanction might vary spatially as a function of a variety of factors, such as the conflict or risk-level in that area. In a study of acceptance of poaching across Norway and Sweden, Gangaas et al. (2013) found acceptance of poaching was highest in areas with both free-ranging sheep and strong hunting traditions (two sources of human-carnivore conflict), suggesting that the risks carnivores posed to livestock and hunting might affect whether individuals would act as guardians. Thus, in addition to measures of enforcement activities (which may be hard to acquire), researchers could use self-reports of ‘perceived norms’ (the extent to which people living in an area believe poaching is common in that area) or subjective evaluations of the likelihood of being sanctioned (caught, prosecuted and severely punished). Such measures could also capture what might be called ‘anti-guardianship’ – that is, a situation whereby local social norms actually encourage the illegal killing of particular species seen as nuisances, pests, or threats to life or property. There is a plethora of psychological theory and research detailing the effects of norms on behavior, including numerous and often context-specific meta-analyses that may provide additional insights (e.g., Poškus 2016; Sheeran et al. 2016); Niemiec et al. 2020; Melnyk et al. 2022).

### Institutional facilitation

Various government bodies create policy that potentially facilitates or inhibits the different sources of human-caused carnivore mortalities. Examples of such policies include bag limits (harvest), level of enforcement (illicit), policies requiring non-lethal measures of livestock protection (control), and in the case of vehicle collisions, speed limits (accidental). Identifying and describing every governmental policy that potentially impacts HCMs is beyond the scope of this paper. The point, for clarity, is that these policies vary spatially (by geopolitical unit) in ways likely to create variation in mortality risk across different mortality sources. Moreover, such laws themselves can provide a ‘signal’ to both hunters and poachers regarding the acceptability of such activities. For example, Chapron and Treves (2016) found that liberalizing hunting seasons or control actions also likely increased the probability of poaching of wolves. In effect, the policy of promoting legalized carnivore killing can send a signal that killing, in general, is more socially acceptable, thereby making illegal killing probable.

## Synthesis and additional considerations

Social science research has increasingly revealed insights concerning the underlying processes that promote both carnivore conservation activities (e.g., reintroduction), as well as some forms of carnivore mortalities (i.e., hunting, poaching). Yet, such studies are rarely spatially explicit. Even where spatially explicit human dimensions research has been conducted, studies have tended to quantify just one of the underlying processes—often characterized as some type of ‘attitude’. Examples include spatially explicit models of attitudes toward species (e.g., Bowman et al. 2004), social/human acceptance (e.g., Behr et al. 2017; Sage et al. 2022), or support for the lethal control of a species (Manfredo et al. 2021). The question, put simply, is how to synthesize insights from across the social sciences to provide better projection of where and why HCMs occur? To that end, we sought to demonstrate how theory from the social sciences could be used to better understand some of the key antecedent processes that promote and inhibit HCMs.

One key challenge in modeling the antecedents of HCMs is how to collect sufficient data to make predictions over relatively large spatial scales. Manfredo et al. (2021) recently detailed a method that may be useful in this regard. They used individual-level self-reports collected surveys across states and counties to estimate *mutualism* and *domination*—two measures that concern people’s perceptions of ideal relationships with wildlife, along with support for the lethal control of gray wolves. Next, they combined and summarized these measures at the county scale and appended data collected by various federal agencies to identify variables correlated with those of interest. Finally, they used modeling to obtain coefficients that were then used to project both a combined measure of mutualism and domination as well as support for the lethal control of wolves across all (> 3,000) counties in the contiguous U.S.

In effect, Manfredo et al (2021) modeled a proxy for one of the four factors (intention) we outlined as critical to understanding spatial variation in HCMs. However, intention is just one of the antecedents impacting HCMs. Additional research is needed to expand upon this effort; specifically, research that seeks to assess and test different measures of each of the four factors (i.e., opportunity, intention, capacity and guardianship) outlined here would be of considerable value—especially if these factors could be related to recorded HCMs at the appropriate spatial and temporal scales. We expect this to be especially challenging for illicit mortalities as those engaged in illicit killing generally attempt to conceal their efforts (Liberg et al. 2020). Recent research attempting to estimate ‘cryptic’ or unobserved poaching is likely to provide useful insights (Liberg et al. 2012; 2020).

Elucidating variation in the other antecedents of HCMs could promote a more accurate picture of *where* and *why* mortalities are more or less likely to arise. We contend that each of the antecedent processes characterized above—opportunity, capacity, intention, guardianship and institutional facilitation—can be usefully characterized across spatial units relevant for large carnivore conservation using various survey methods (with careful attention to sampling). Moreover, the procedure outlined by Manfredo (2021) could be used to project scores for each of the five antecedents in unsampled units (assuming scores correlate with existing data sources). The result of such an effort would be a combination of measures and projected scores that could be used to explain where various types of HCM are more or less likely to occur.

Over time, we anticipate that careful model specification and testing will allow us to improve projections of where different kinds of mortalities will occur. Furthermore, the ability to more accurately map different types of anthropogenic risks to carnivores could be used to guide policy-making, including, for example, identifying appropriate places for species restoration efforts, refining harvest management across jurisdictions with different types of anthropogenic risks, or determining where to focus enforcement efforts (e.g., Benson et al. 2023). Moreover, specifying these processes a priori allows for testing of hypothesized causal mechanisms—thereby offering a richer understanding of the underlying phenomena. This understanding is aided by the recognition that at least some of the proximate processes that precede any instance of an intentionally-caused mortality exist entirely in the minds of humans, and therefore, require integration of theory and insights from the social and behavioral sciences.

We recognize that sources of mortality along with the importance of various antecedent processes are likely to vary from species to species and across different sociopolitical units (e.g., in states where hunting mountain lions is regulated versus those in which it is unregulated, like Texas; Elbroch and Harveson 2022). A recent analysis of carnivore occupancy over time in Europe concluded increased habitat suitability for LCs was most likely a function of changes in forest cover and decreased human population density (Cimatti et al. 2021). Both changes (i.e., forest cover, population density) are, in part, a result of myriad policy decisions that led to long-term changes. Similarly, other studies have found evidence that carnivore conservation success at the country level are tied to factors such as the mechanization of agriculture and improvements in basic infrastructure (Bruskotter et al. 2017). Consideration of such social changes could also yield new insights and improve our understanding of the factors affecting the success or failure of carnivore conservation efforts.

The likelihood of variation across sociopolitical units raises the issue of appropriate scale for these types of analyses. Scale is a significant challenge for multiple reasons. First, because people tend to be extremely unevenly distributed, the ability to reach adequate sample sizes to make reasonable inferences about the population living within an area will vary considerably and may be particularly challenging in the least populated areas—which are often the very places we expect to find large carnivores. Second, processes that yield an effect at one scale have been known to produce different effects—sometimes even reversing directionality—at different scales. While we do not have a priori reasons to expect this sort of sensitively to scale for the processes we have described, its possibility suggests careful attention to scale is warranted. Finally, the method of projecting from a sampled set of units to a broader population used by Manfredo and colleagues (2021) relies on publicly available data that may not exist in certain countries or may be aggregated at scales that are too to be useful. For these reasons, we expect determining appropriate scale and finding data at that scale to be a significant challenge, particularly for areas of less dense human habitation or with less data coverage.

A careful reader might point out that the conditions in any specific geographic area will vary because people are mobile. Thus, for example, an individual residing in a large, urbanized setting may choose to travel to less densely populated area to hunt. Although this undoubtedly creates some error when assigning values to spatial units based on residency, we believe the effects of such error, in most cases, will be minimal. We base this assessment on data showing that, in one of the world’s most mobile societies (the United States), data indicate that, on average, adults spend 80–90% of their time either in the home or at work (Ortiz-Ospina et al. 2020), and commute times average about 27 min one-way. Moreover, because of the costs involved in traveling for the purposes of hunting—both financial and opportunity costs—we anticipate most individuals will attempt to minimize travel distance. Thus, location of residence should provide an adequate proxy for the location of HCMs and antecedent processes.

While the focus of our efforts was on individual-level attributes that would tend to promote or inhibit behavior resulting in HCMs, we also acknowledge the importance of group-level factors (e.g., culture, institutions) that have been the focus of other traditions in the literature on human-wildlife conflict. For a toehold into the insights provided by such work see, Skogen et al. (2019), Massarella et al. (2021), Toncheva and Fletcher (2021) and Pooley et al. (2021). In particular, work from these disciplines is useful for understanding underlying causes that might lead members of certain groups to be more or less likely to manifest the types of behavioral antecedents we discuss. We hasten to add that, because our focus is predicting where HCMs will occur, our efforts necessarily emphasized proximate rather than ultimate causes of such behavior–thus, our focus on individuals.

Though our initial efforts focused on explaining variation in processes that promote HCMs in North America, we expect the antecedent processes we describe—i.e., opportunity, capacity, intention, guardianship and institutional facilitation will generalize to many other contexts. Indeed, it is hard to imagine a context where rates of illegal killing, for example, are not affected by the collective capacity to a population to carry out such acts. However, while we anticipate these factors to be broadly generalizable, we also anticipate great variation across contexts in the ultimate causes of HCMs.

Relatedly, although Table 2 connects each of the types of HCM we discuss to novel processes, we recognize that many jurisdictions may have existing means to estimate and even project the number of mortalities due to accidents, harvest and control actions with relative accuracy. Using roads as a proxy for vehicle strikes, for example, is likely sufficient for most instances of estimating accidental HCMs. Thus, much of the novelty herein is in making projections about where illicit mortalities are likely—a subject that has been the source of much debate in recent years (e.g., Louchouarn et al. 2021, Santiago-Avila and Treves 2022).

Importantly, our effort was not aimed at exhaustively cataloging the ways human institutions affect carnivore conservation; rather, we attempted to capture the most proximate and effectual ways in which humans alter the risk of HCMs. Thus, while we present an approach that we believe will be useful for most LCs, its use will require some adaptation to make it suitable for each specific, species-by-sociopolitical context. Such adaptations would do well to pay attention to issues of scale, jurisdictional boundaries, local hunting customs, as well as the economic value of the species in question—each is potentially an important part of the systems that promote or inhibit HCMs.

## Data Availability

Data not available. No data were used in the preparation of this manuscript.

## Footnotes

1 A related type of accidental mortality occurs due to mistaken identity (e.g. when a hunter kills an animal believing they were killing a different type of animal). In practice, this type of mortality involves that same antecedent processes as any harvest (see Table 1), and therefore, we do not attempt to differentiate it from normal harvest mortality.

2 Unlike an encounter, capacity is not required, but rather increases the probability that one intent on killing a particular type of animal will successfully find and dispatch that animal.
